# The distillation method: A novel approach for analyzing randomized trials when exposure to the intervention is diluted

**DOI:** 10.1111/1475-6773.14014

**Published:** 2022-07-19

**Authors:** John L. Adams, Anna C. Davis, Eric C. Schneider, Michaela M. Hull, Elizabeth A. McGlynn

**Affiliations:** ^1^ Center for Effectiveness and Safety Research Kaiser Permanente Pasadena California USA; ^2^ Department of Health Systems Science Kaiser Permanente Bernard J. Tyson School of Medicine Pasadena California USA; ^3^ Quality Measurement and Research Group National Committee for Quality Assurance Washington District of Columbia USA; ^4^ Health Plan and Hospital Quality Kaiser Permanente Oakland California USA

**Keywords:** biostatistical methods, internal validity, learning health system, participant responsiveness, randomized controlled trial

## Abstract

**Objective:**

To introduce a novel analytical approach for randomized controlled trials that are underpowered because of low participant enrollment or engagement.

**Data Sources:**

Reanalysis of data for 805 patients randomized as part of a pilot complex care intervention in 2015–2016 in a large delivery system. In the pilot randomized trial, only 64.6% of patients assigned to the intervention group participated.

**Study Design:**

A case study and simulation. The “Distillation Method” capitalizes on the frequently observed correlation between the probability of subjects' participation or engagement in the intervention and the magnitude of benefit they experience. The novel method involves three stages: first, it uses baseline covariates to generate predicted probabilities of participation. Next, these are used to produce nested subsamples of the randomized intervention and control groups that are more concentrated with subjects who were likely to participate/engage. Finally, for the outcomes of interest, standard statistical methods are used to re‐evaluate intervention effectiveness in these concentrated subsets.

**Data Extraction Methods:**

We assembled secondary data on patients who were randomized to the pilot intervention for one year prior to randomization and two follow‐up years. Data included program enrollment status, membership data, demographics, utilization, costs, and clinical data.

**Principal Findings:**

Using baseline covariates only, Generalized Boosted Regression Models predicting program enrollment performed well (AUC 0.884). We then distilled the full randomized sample to increasing levels of concentration and reanalyzed program outcomes. We found statistically significant differences in outpatient utilization and emergency department utilization (both follow‐up years), and in total costs (follow‐up year two only) at select levels of population concentration.

**Conclusions:**

By offering an internally valid analytic framework, the Distillation Method can increase the power to detect effects by redefining the estimand to subpopulations with higher enrollment probabilities and stronger average treatment effects while maintaining the original randomization.

**What is known on this topic**

- Randomized trials to test new care delivery models are often underpowered due to lower‐than‐expected participation among the randomized intervention population.
- In these scenarios, intent‐to‐treat analyses may produce false‐negative results, but as‐treated analyses are likely to be biased by selection effects.

**What this study adds**

- This study introduces a novel approach for analyzing trials when a large proportion of patients randomized to treatment received an insufficient amount of the intervention.
- The Distillation Method can increase power by redefining the estimand to focus on patients with a higher probability of engaging with and benefiting from the intervention while retaining randomization status.

## 1 INTRODUCTION

By the time they are completed, randomized trials to test the effectiveness of new care delivery models are often underpowered.^1^ Overly optimistic estimates of effect sizes can lead planners to underestimate necessary sample sizes (resulting in lower actual statistical power).^2^ Sample sizes can also be undermined when participation is lower than expected.^3^, ^4^, ^5^ Even among enrolled populations, uneven delivery of the intervention or poor subject engagement can reduce exposure to the intervention. These patterns of variable participant responsiveness are a special class of heterogeneity. Within any randomized sample, patients in the intervention group will vary in their likelihood of taking up the intervention and their engagement with it.

**FIGURE 1:**
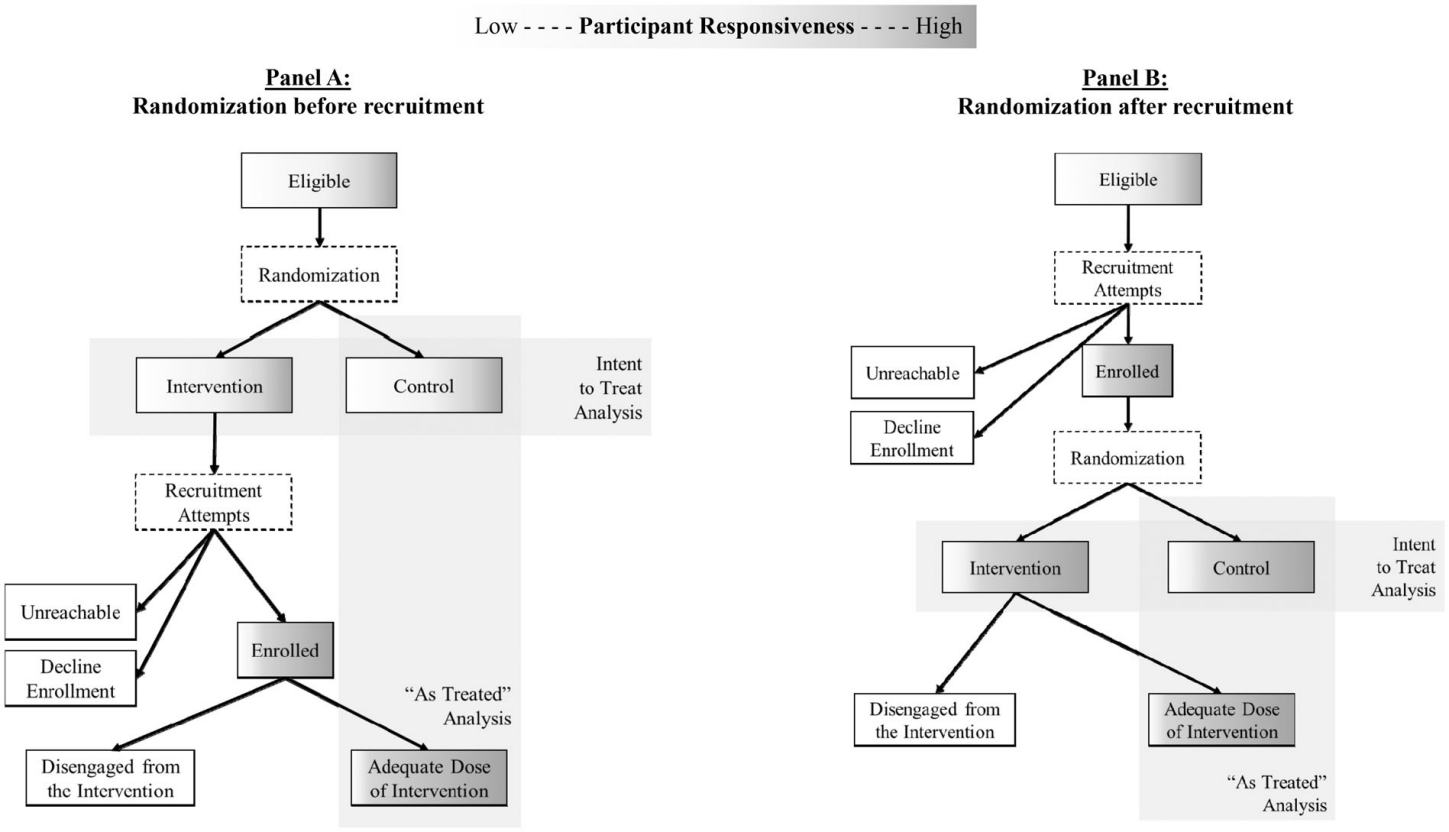
Dilution of the intervention in randomized evaluations of health care innovations. In any eligible population, there is heterogeneity in the probability of uptake/engagement with a given intervention (represented by gradient shading). Dilution can arise throughout the trial implementation process, whether randomization takes place before recruitment (Panel A) or after recruitment (Panel B). Comparing the subpopulation that is ultimately treated to the full control group in “as‐treated” analysis introduces bias when those who take the treatment are different from those who do not take it for any reason. The Distillation Approach uses the patient groups from the intent‐to‐treat stage and distills both groups in an equivalent manner, based on the predicted participant responsiveness rather than observed uptake or engagement (which is available for the intervention group only).

The problem of insufficient take up of the intervention within the treatment group of an RCT can be thought of as “dilution” of the trial because the intervention effect is diluted in intent‐to‐treat (ITT) trial analyses by the presence of patients in the treatment group who either did not receive or only partially received the intervention. In the case of health care innovations that are targeted to specific patient populations, a diluted ITT estimate might cause innovators to mistakenly discard an intervention based on false‐negative results, although it is actually effective for a narrower subset of the patient population originally selected. Figure 1 compares trial processes when randomization is completed before (Panel A) or after (Panel B) participants are recruited into an RCT; in both scenarios, dilution of the treatment group can occur, although it is more problematic when participants are randomized *before* recruitment. In many randomization‐before‐recruitment trials, the control group is not actively recruited, but their usual care and outcomes serve as the comparator. Even when study participants are randomized *after* recruitment, dilution can occur through low engagement or inadequate intervention delivery. For example, in an RCT that requires some period of in‐person counseling or another follow‐up, there may be variation among participants in their attendance of those sessions.

When design and implementation failures occur in RCTs, evaluators frequently default to analysis techniques that introduce new biases. “As treated” analyses (Figure 1) compare the full randomized control group to the subset of the treatment group that received (an adequate dose of) the intervention, but this approach effectively eliminates the benefits of randomization if there is any mechanism of channeling by the patient or by other agents (i.e., self‐selection bias).^6^ Propensity score analyses, which match each treated patient with a control patient having similar characteristics, attempt to overcome this systematic difference.^7^ However, the differences within the treatment group between those who participate and those who do not may be driven by unmeasured factors like motivation, self‐efficacy, perception of potential benefit, and perceived participation burden.^8^ Propensity scoring methods cannot match these unmeasured factors.

In this paper, we describe a new analytic approach for RCTs that are affected by dilution. Our approach, which we call the “Distillation Method,” addresses the issue of dilution by capitalizing on the observation that responsiveness (the likelihood that a participant takes up or engages in the intervention) is heterogeneous within the treatment group of a randomized trial and may be positively correlated with the magnitude of the treatment effect. This method refocuses the analysis on a new estimand and yields information about the effect of the treatment in a narrower study population that has been trimmed to exclude those with a lower *probability* of uptake from both the treatment and control arms. The approach offers a complementary analysis to ITT results, in any trial where participation, engagement, or dose/fidelity was inconsistent within the treatment arm. It is designed to allow evaluators to restrict the analysis of a randomized study to a subpopulation with larger average treatment effect sizes by excluding patients with a lower probability of participation *in a way that retains the benefits of the original randomization*. Under the right conditions, the Distillation Method can improve statistical power to measure the intervention effect.

## 2 OVERVIEW OF THE DISTILLATION METHOD

The Distillation Method involves a three‐stage analysis, which we describe generically here. We then present first a case study in which we reanalyze real data from an RCT that was randomized before recruitment and experienced dilution due to low enrollment among patients randomized to the intervention group. This is followed by a simulation study that characterizes the properties of the approach.

In the first stage of the Distillation Method, we predict participation in the intervention using a machine learning approach and available predictors drawn only from the baseline period. In this stage, we build predictive models of participation within the intervention group. Covariates in the first stage model can include demographics, comorbidities, risk scores/severity scores, condition‐specific measures of disease or severity (e.g., HbA1c for patients with diabetes), utilization during the baseline period (e.g., acute care, ambulatory care, preventive care, prescriptions), measures of access or engagement with care (e.g., primary care continuity), measures of adherence (e.g., medication adherence), insurance status/coverage information, data on social/nonmedical needs, and so on. Given that causal interpretation is not the primary purpose of the stage one model, it is acceptable to allow the machine learner maximum flexibility to fit a model that leverages any available baseline predictors. Performance of the first stage model is assessed using typical summary quality of prediction measures (e.g., AUC, C statistics, etc.), as well as measures of the relative importance of the predictors.

The goal of stage one is to identify a subset of the randomized sample with a higher predicted probability of participation. The Distillation Method is an ITT estimator for a specific subpopulation that is more likely to participate in the intervention. It is not an estimator for the original population average treatment effect. The Distillation estimand can be thought of as the population average treatment effect for a trial with more targeted eligibility criteria.

The second stage is the crux of the methodologic innovation. Using the results of the stage one models, we subset both the randomized intervention and control groups so that they are distilled in an equivalent manner to a subset of the overall study population with a higher probability of participation. This distillation step reduces the total sample size and removes both treated and untreated patients who had a low probability of uptake. The exclusion of treated patients with a low probability of uptake is necessary to retain the unbiased properties of the randomization. This stage creates a counterfactual where people are included in the control group if they would have also been more likely to participate in the intervention. Different cut points in the degree of distillation can explore different trade‐offs between concentration and reduced sample size.

Finally, in the third stage, we re‐evaluate the intervention for effectiveness to test whether the informed subsetting approach identifies a population where the intervention effect is statistically significant. These third‐stage models for intervention effectiveness should use the most appropriate statistical methods for the outcome of interest (e.g., Poisson for utilization or gamma models for cost outcomes). In many cases, a difference‐in‐differences approach will be appropriate for this stage of analysis; the Distillation Methodology is agnostic to the choice of outcome model. The outcome model is refit to increasingly distilled/concentrated subsets of the original data defined by levels of the predicted probabilities from the stage one intervention participation model. Because these sequential tests are nested and prespecified, there is limited risk of multiple comparisons problems.

### 2.1 Assumptions and limitations

The Distillation Method has two additional assumptions beyond the usual RCT assumptions. First, it requires that there is a positive correlation between treatment uptake and the magnitude of the treatment effect. Second, there must be at least one pretreatment predictor of treatment uptake. If there is no correlation between the probability of uptake and treatment effect or there are no predictors of uptake, the estimands of the Distillation estimate and the original ITT estimate will be the same. The Distillation Method requires that the original study was randomized, and data on intervention uptake or participation must have been retained for each patient in the intervention group. Like any method for analyzing an RCT, the results of the analysis relate to the intervention as implemented. Appendix A offers an additional discussion of the method and its assumptions and limitations.

## 3 CASE STUDY: APPLYING THE DISTILLATION METHOD TO A COMPLEX CARE RCT

### 3.1 Methods of the case study

#### 3.1.1 Data assembly and study population

This case study is an application of the Distillation Method using data from a multidisciplinary complex case management intervention that was pilot tested in an RCT within Kaiser Permanente Southern California during 2015–2016. Adult members who were predicted to have high costs in the coming year (using a proprietary risk prediction algorithm), were age 18 or greater, resided in one county‐based service area within the Southern California region, and had current Kaiser Permanente membership were randomly assigned in December 2015 to the pilot study. In total, 805 patients were randomized: 404 to the intervention arm and 401 to the control arm. Intervention group patients were recruited after randomization.

The intervention team comprised registered nurse case managers, care navigators, a social worker, and a physician. The intervention included case conferences, proactive intensive case management, home visiting, and coordination and linkage with other programs and services for which each patient was eligible, including health care, community‐based, and social needs services. Program participants were discharged from the program based on clinician assessment of ongoing needs.

Members were included in this analysis if they were randomized as part of the pilot intervention. We used intervention tracking logs from the pilot to identify each member's date of randomization, randomization status, and—for those randomized to the intervention group—the date of enrollment for those who consented to participate (enrolled). Among the 404 patients randomized to the intervention group, 261 (64.6%) enrolled in the intervention. The main reasons for nonenrollment were that the member could not be contacted or the intervention was declined. The average duration of intervention services for those patients who were successfully enrolled was 4.6 months (ranging from 6 days to 1.4 years).

Using the unique patient identifiers from the intervention roster and the dates of randomization, we assembled a dataset from Kaiser Permanente's Integrated Data Repository and Geographically Enriched Member Sociodemographic datamarts. These records reflect information about member demographic characteristics, comorbidities, utilization, costs, block‐group level census data, membership history, and a range of proprietary risk scores. All baseline characteristics were defined using the 12‐month period prior to the date of randomization.

For each member, the date of randomization defined the start of the follow‐up period. We measured outcomes in two 12‐month follow‐up periods; the first follow‐up period included the period of intervention delivery (enrollment to month 12), and the second period was the subsequent year (months 13–24). Outcome measures for the intervention aligned with those originally proposed for the initial evaluation. Specifically, we captured utilization and cost data for the following categories of care: inpatient admissions, inpatient days (excluding observation days), treat and release emergency department visits, and ambulatory care visits (outpatient primary and specialty care); we also captured data on the total cost of care (including ancillary services). The use of these secondary data for this study was approved by the Kaiser Permanente Southern California Institutional Review Board.

#### 3.1.2 Statistical methods

In the first stage of the Distillation Method analysis, we predicted intervention uptake within the randomized intervention group, using enrollment (ever/never based on the intervention roster) as the outcome measure. Using the R package GBM version 2.1.8, we employed Generalized Boosted Regression Models (GBM) and all available baseline variables to predict enrollment. GBM is a machine learning tool that is used by statisticians in evaluation studies. We used 10‐fold cross‐validation, a maximum of 10,000 trees (the optimum number was selected by the model using the cross‐validation [resulting in 2187 trees]), a maximum interaction depth of 2, a minimum leaf size of 8, and a learning rate of 0.001. Although the outcome itself was binary, the resulting prediction is a probability between 0 and 1.

In stage two, we established five levels of population distillation based on predicted probabilities of intervention uptake for both the intervention and control groups, derived from the stage one model estimates. As the population definition was increasingly distilled, the total sample size decreased, and the remaining sample (in the treatment group) became more concentrated with patients who received the intervention.

Our outcome analyses for costs and utilization included both the baseline and follow‐up period outcome values and an indicator for the study period. This approach, a general “difference‐in‐differences” analysis, allows us to look at the difference between the time periods in the outcome variable by comparing the baseline period to each of the follow‐up periods for the intervention versus the control. Thus, for each outcome variable we have a series of one‐year results. We used two sequential follow‐up periods rather than accumulating the follow‐up data into a single time period to give us insights into delayed benefits and/or treatment decay over time.

To model intervention outcomes, we used generalized linear mixed‐effects models (GLMM) using the R package lme4 (version 1.1‐18‐1) with offsets to account for months of membership in the period of interest. We used a gamma distribution for costs and a Poisson distribution for encounter counts. Our main analysis is the single‐year change from the baseline period to each of the follow‐up period years.

The encounter count models were Poisson models of the form:

$$\ln\left(\lambda_{\mathrm{it}}\right)=\beta_{0i}+\beta_{t}∙I_{t=\text{post}}+\beta_{\text{intervention}}∙I_{\text{intervention}}+\tau ∙I_{t=\text{post}}∙I_{\text{intervention}},$$

$$\beta_{0i}\sim \text{Normal}\left(\beta_{0},\sigma^{2}\right).$$

$\lambda_{\mathrm{it}}$ is the person‐time period event rate. Where *i* indexes the person and *t* is the time period. $\beta_{0i}$ is the intercept for the *i*th person. This is a normally distributed random effect. This also adjusts the standard errors for person level clustering. $\beta_{t}$ is a common time effect. $\beta_{\text{intervention}}$ is an effect unique to the intervention group across both the baseline and follow‐up periods. With randomization this has an expected value of zero but may be different from zero in finite samples. τ is the treatment effect. This is a generalized difference of differences model. The gamma cost model has a similar dependent variable structure but assumes a gamma distribution with a log link. Both models use offsets for the person level exposure months in the time periods. These treatment effect parameterizations have an approximate percentage change interpretation.

### 3.2 Results of the case study

#### 3.2.1 Predicting intervention uptake

Our model predicting intervention uptake performed well with an AUC of 0.884. The most influential predictors in the model are shown in Appendix B Figure B.1. Predictors with high relative influence included several risk scores from the Verisk Health/DxCG proprietary software,^9^ as well as measures capturing baseline year utilization and health status.

#### 3.2.2 Evaluating intervention effectiveness with the distillation innovation

**FIGURE 2:**
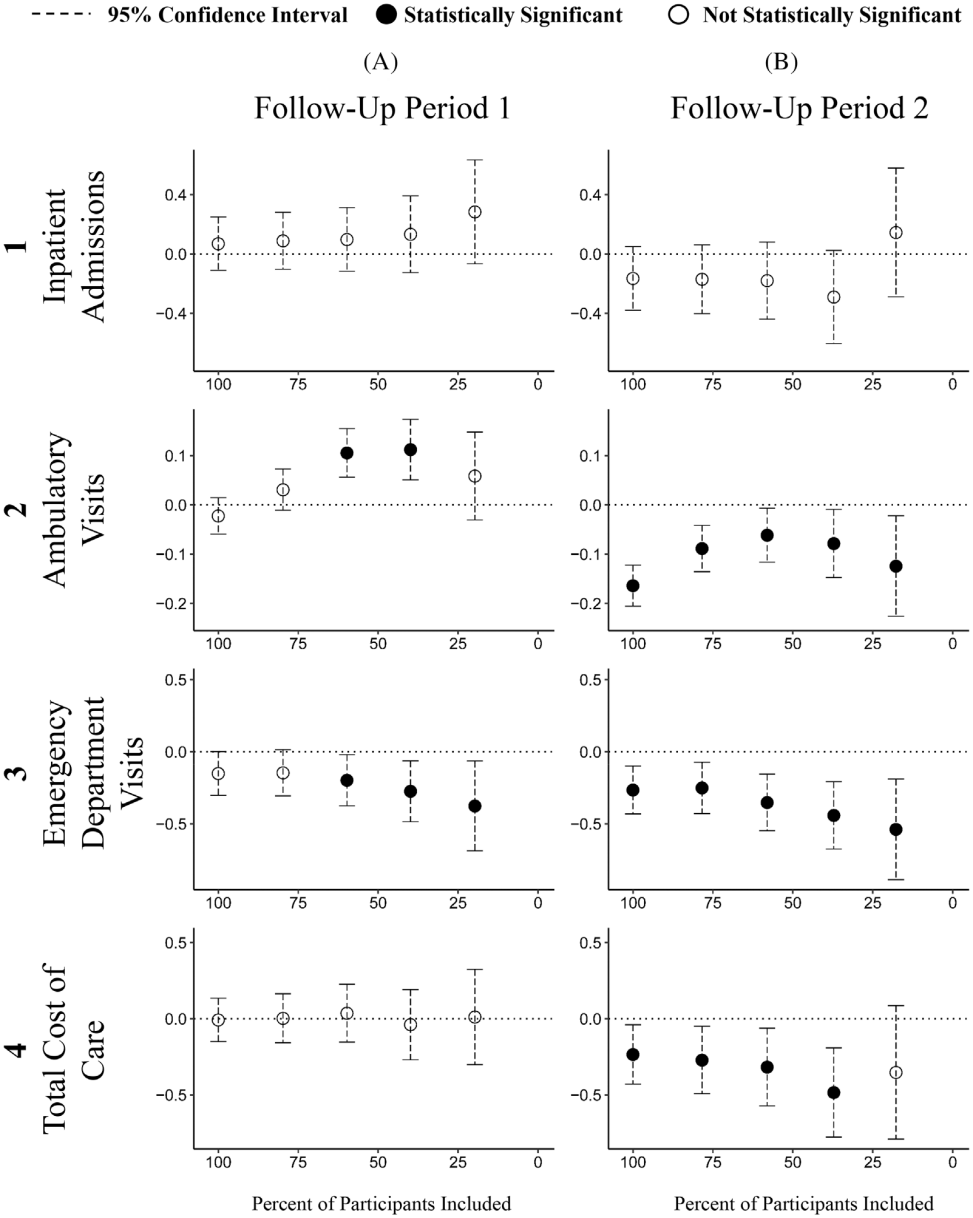
Utilization‐ and cost‐based measures of intervention effectiveness at five levels of population distillation for an RCT of a complex case management intervention. The *x*‐axis in each chart within the grid represents the level of distillation based on the predicted probability of enrollment from the stage one model, displayed as the percent of participants included in the analysis. The concentration of the population increases from left to right in each chart. Columns A and B are the two follow‐up periods compared to baseline; Rows 1 through 4 are the outcomes. Statistically significant results (in which the change variable is significantly different from zero at the *p* = 0.05 level) are shown with solid black dots, while nonsignificant results are unfilled dots; 95% confidence intervals are shown with dashed lines.

Results of the intervention effectiveness analysis are shown in Figure 2. This figure presents a grid of results, with the two follow‐up time periods in columns A and B, and outcome variables arrayed in rows one to four: the utilization‐based measures are in rows one to three, and results for total health care costs are in the fourth row. The concentration of the population increases as we move from left to right in each chart; the first result in each chart is the standard evaluation ITT estimate with 100% of the data included and patients analyzed as randomized. With increasing concentration of the study population, the confidence bands get larger as expected because of the reduction in sample size.

As shown in the first row of Figure 2, the change in the number of hospital admissions is never statistically significant; however, during the second follow‐up period (column B) the point estimates directionally indicate a decrease at most levels of population distillation. Changes in count of ambulatory care visits (second row) indicate that the intervention was associated with an increase in outpatient utilization during the first follow‐up period at a moderate level of population distillation. There is a significant decrease in outpatient utilization at all levels of distillation during the second follow‐up period. Counts of emergency department visits (third row) are significantly lower at moderate levels of distillation during the first follow‐up period and significantly lower at all levels of distillation during the second follow‐up period.

During the first follow‐up period, there were no significant changes in total costs (Figure 2, column A, fourth row) at any level of distillation; this follow‐up period included intervention delivery. However, in follow‐up period two (Figure 2, column B, fourth row), our results indicate a statistically significant decrease in total costs at most levels of distillation. During the second follow‐up period, when the population is at its most distilled, the confidence band on the total cost estimate widens enough that the result is no longer statistically significant.

## 4 DISTILLATION OF TREATED CASES: A SIMULATION STUDY TO EVALUATE THE METHOD

### 4.1 Methods of the simulation study

To further describe the relationships that govern the Distillation Method, below, we present one case from a simulation of the method to demonstrate the features that drive the potential usefulness of this method. Appendix A presents additional results of the simulation considering a wider range of values.

In this example from the simulation, we assume that 40% of the study population benefits from the treatment and that 40% participate in the treatment. The relationships in the simulation are generated by three correlated normal random variables: the outcome, the treatment effect, and the prediction of participation. Conceptually this outcome would correspond to a log‐dollars cost model or a normal approximation of a log utilization model. The treatment effect relationship assigns a treatment value of Taumax to the subjects in the top 40% of the treatment effect normal variable. Similarly, the top 40% who are predicted to participate are assigned to the enrolled group. The underlying relationships are controlled by the correlations between the normal random variables. In the example, the correlation between treatment effect and participation is 30%, between treatment effect and prediction of participation is 80%, and between participation and prediction of participation is 80%. Values of Taumax from 0 to −0.5 are presented for illustration. A Taumax of −0.5 would correspond to approximately a 50% reduction in cost or utilization based on receipt of the treatment. These favorable relationships were selected to make the properties of the Distillation Method clearer. For a complete discussion of the power simulation in its full factorial form, see Appendix A.

### 4.2 Results of the simulation study

**FIGURE 3:**
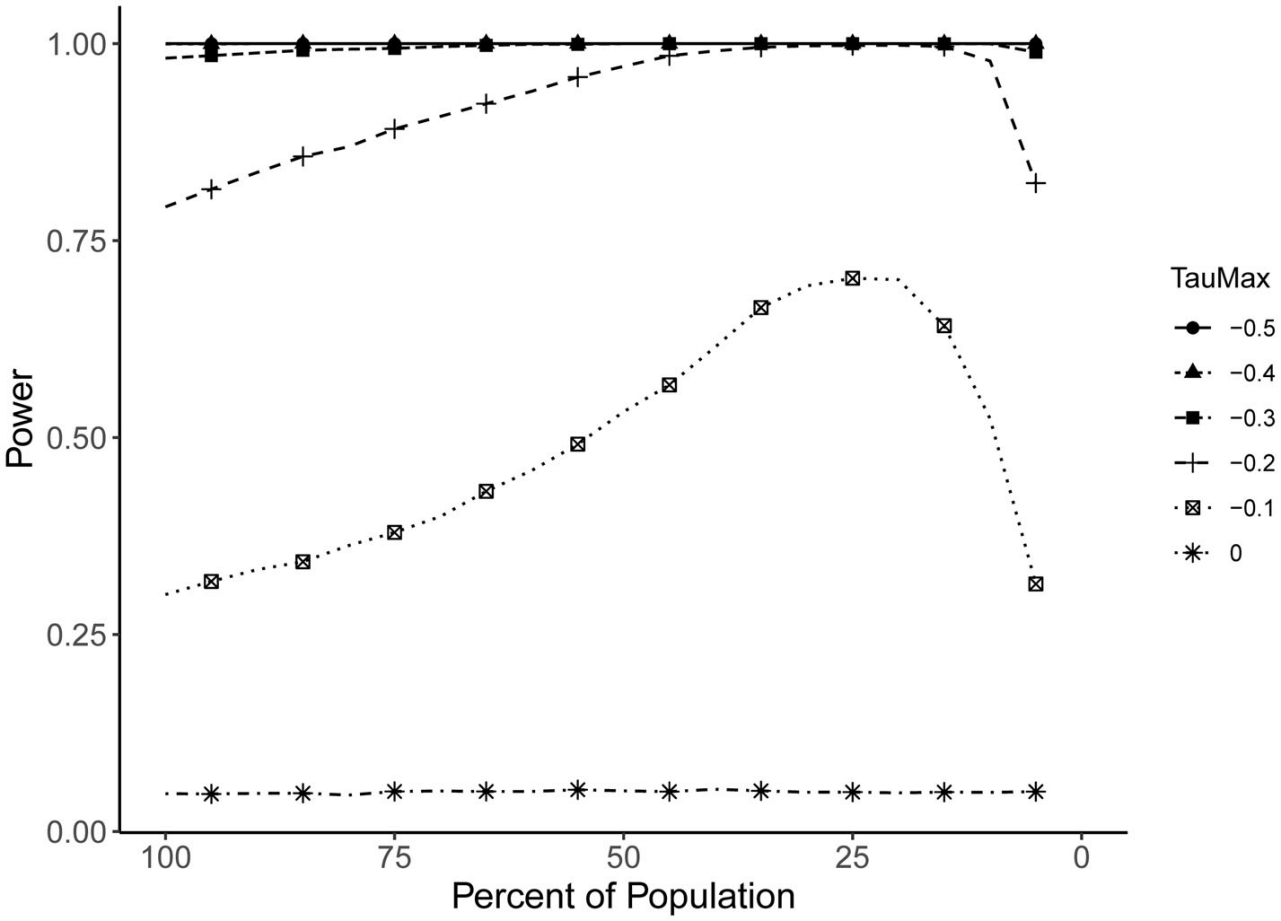
Power as a function of maximum treatment effect size, distillation (a percentage of the population), and enrollment. The chart shows changes in power (*y*‐axis) as distillation is increased (*x*‐axis, displayed as the percentage of participants included in the analysis). In this scenario from the simulation, the correlation between treatment effect and participation is 30%, between treatment effect and prediction of participation is 80%, and between participation and prediction of participation is 80%. Taumax (plotted lines) represents treatment value. Values of Taumax from 0 to −0.5 are presented for illustration. A Taumax of −0.5 would correspond to approximately a 50% reduction in cost or utilization based on receipt of the treatment.

Figure 3 illustrates the properties of the method. First, consider Taumax = 0, which is the null case where the intervention is not effective for anyone. In this case, we would expect the power to be 0.05 corresponding to the type I error rate of the test. The method does not increase power because there is nothing to distill. At the opposite end, consider Taumax = −0.5. There is no additional power available, nor is there any additional power needed at these large effect sizes. The results are similar when Taumax = −0.4 and − 0.3. At Taumax = −0.2, we start to see some of the desired distillation effects. The most interesting and important case is the Taumax = −0.1 effect size, where the full data power of approximately 30% is distilled to a power of nearly 70% when the study population is narrowed to just 25% of the full sample.

One of the goals of the method is to control the type I error rate or false‐positive results. The Taumax = 0.0 line in Figure 3 is encouraging since it approximately maintains the 0.05 test level across the range of distillation. Note that these are not independent tests since the subpopulations are nested, thus minimizing multiple comparison effects. Furthermore, because this analysis strategy is prespecified, it does not constitute an undisciplined search for significant results. The simulation method can easily be modified to quantify the multiple comparison effects under various assumptions if this is a concern.

## 5 DISCUSSION

Achieving a comprehensive evidence base for trials of innovative models of health care delivery is among the central goals of a learning health system that generates real‐world evidence.^10^, ^11^ To maximize the knowledge generated from intervention trials in real‐world settings, evaluators could benefit from additional analytic tools that overcome the implementation challenges of conducting randomized trials in a learning health system. In this paper, we describe the Distillation Method, provide an illustration of its application to a real‐world trial, and offer a simulation to further highlight its potential uses.

Rigorous randomized evaluation designs are preferred to produce unbiased estimates of intervention effect when testing new models of care, but intervention trials—especially involving complex health care model interventions—are difficult to carry out. The alternatives, observational studies, and those with weaker study designs such as preperiod versus postperiod comparisons are prone to many biases and rarely enable reliable causal inference.^12^, ^13^

One reason that randomized study designs are preferred is that they offer strong protection against incorrectly concluding that an ineffective intervention works.^12^, ^13^ This is a good preference as the implication of false‐positive results could be propagating interventions that do not work. However, when a randomized trial produces null results, evaluators and program designers may be too quick to accept that the program was ineffective. In reality, a trial that produces null results may be explained by any number of problems with trial design, recruitment and enrollment procedures, or intervention delivery, rather than or in addition to (in)effectiveness of the intervention.^2^

In the implementation literature, low uptake of or low engagement with an intervention is often labeled “participant responsiveness” and can include actions by patients, as well as by the people delivering the intervention.^14^ The issue of unequal uptake/participation in health care services is observed by researchers in many disciplines and studies of different types of services. Notable recent RCTs that experienced dilution include the Oregon Experiment (low enrollment) and the trial of the Camden Coalition's hotspotting model (low engagement after enrollment).^15^, ^16^ One way to limit dilution is to randomize patients after they have been recruited. In this approach, dilution due to low uptake is limited or completely absent because the patients who are randomized have already agreed to participate. However, systems leaders may find this approach to randomization unpalatable or infeasible. Furthermore, it does not protect against dilution related to insufficient intervention exposure (as was the case in the hotspotting model RCT).

The Distillation Method is a novel analytic approach for evaluating data from RCTs that were diluted by suboptimal enrollment or engagement of the population randomized to the intervention group. The method may be most relevant for analyzing RCTs of health care or social needs care models, where interventions are typically targeted to specific patient populations, and where receipt of the intervention is reliant—at least in part—on patient uptake and engagement. In our case study analysis, dilution was substantial, with only 64.6% of the treatment group enrolling in the intervention. Using the Distillation Method, we concentrated the analysis on patients with a higher probability of uptake and identified significant treatment effects of the intervention on ambulatory visits, emergency department visits, and total cost of care, particularly in the second follow‐up year after the intervention period was complete.

The key motivation for the Distillation Method is that it can capitalize on one form of channeling by indication, in which providers' and patients' intuition about potential benefit from an intervention drives enrollment or engagement of individuals into treatment.^17^ While such channeling is often problematic in observational evaluation analyses, the Distillation Method can turn it into an advantage. If those who are successfully recruited are no different than those randomized to the intervention (and the assumption that uptake/engagement is correlated with treatment effect is not upheld), the Distillation Method will just reduce sample sizes with no corresponding increase in intervention effects. But if the successfully recruited participants have above‐average benefits from the intervention, this approach will increase the average treatment effect size while decreasing the sample size. This approach can turn the most common threats to validity in “as‐treated” analysis (selection biases) into a power advantage by changing the estimand; the Distillation estimates are unbiased for the new quantity of interest.

Extensions to this approach could be tested in future studies. The method presented here is an example of a class of methods that capitalize on the heterogeneity of treatment effects to identify subpopulations where an intervention is most effective. One extension is to leverage alternative approaches to selecting the distillation thresholds in stage two of the method when the nested population subsets are defined. An intriguing possibility is to distill the population by excluding patients from both extremes of the stage one model results (both very high probability of uptake and very low probability of uptake). This approach might make sense in cases where patients who are highly likely to engage with the intervention are also unlikely to benefit from the treatment (e.g., if their needs are so intense/extreme that a case management intervention is unlikely to help them).

Some especially complex interventions may be prone to several “voltage drops”^18^ that create dilution, as described in Figure 1. The Distillation Method can be flexibly extended to address these subsequent stages of intervention uptake/engagement or other dimensions of the implementation fidelity framework. For example, in our case study of a complex case management RCT, we modeled enrollment as a dichotomous outcome in stage one of the Distillation Method, but the duration of enrollment ranged from 6 days to 1.4 years. Alternate dichotomous definitions of participant responsiveness (e.g., receiving a minimum duration of intervention in days or hours of intervention services) might produce a stronger concentration of treatment effects. The distillation step of the novel strategy can be adapted to use a continuous measure of participation in the first stage (e.g., the actual number of hours of intervention) or repeated with alternate or compound definitions of responsiveness. Finally, interventions that match patients to particular treatment arms can be evaluated with a minor extension of the method by using compound definitions of eligibility that combine enrollment and treatment arm to yield arm‐specific effect estimates.

Ultimately, the Distillation Method will be useful under a specific set of conditions. It is not an estimator for the original population average treatment effect, and as such, may be less useful when an intervention is offered to all comers or mandated. In those situations, the best estimate of the average treatment effect assumes that dilution will occur. Nevertheless, the Distillation Method will have utility in many health care RCTs when implementation fidelity is inconsistent, and eligibility criteria can be molded to select the ideal target population. The Distillation Method offers a new way to examine evaluation results in an internally valid framework building upon a randomized evaluation design. This approach offers an appealing and straightforward alternative to some of the less valid post hoc analyses that are tempting when a trial is dilute, such as “as‐treated” analyses.

The Distillation Method was conceived as a salvage strategy to rescue false‐negative results from the “file drawers”^19^ of the evaluation community writ large, but it may be planned proactively as a complementary analysis to the ITT with the goal of understanding the heterogeneity of treatment effects. The Distillation Method can be conceptually thought of as redefining eligibility criteria for the original RCT to the subpopulation with a greater likelihood of enrolling or engaging. This may reduce the amount of effort spent on interventions relative to the yield in terms of outcomes. An exploration of Distillation estimates within populations with higher uptake probability and larger average treatment effects may even allow innovators to identify treatment group subsets where there is a higher return on investment, using such targeting to increase the impact of interventions for providers and the populations they are managing.

## FUNDING INFORMATION

This work was supported by the Commonwealth Fund, a national, private foundation based in New York City that supports independent research on health care issues and makes grants to improve health care practice and policy. The views presented here are those of the author and not necessarily those of the Commonwealth Fund, its directors, officers, or staff.

## Supporting information


**Data S1.** Supporting information.

**Data S2.** Supporting information.
