# Supplementary material for: The distillation method: A novel approach for analyzing randomized trials when exposure to the intervention is diluted
Source: Health Serv Res. 2022 Jul 19;57(6):1361–9. doi: 10.1111/1475-6773.14014 (PMC9643092; doi:10.1111/1475-6773.14014)
Supplement: Supplementary file 1 — Data S1. Supporting information. [file HESR-57-1361-s001.pdf]

The Distillation Method: A novel approach for analyzing randomized trials when exposure to the intervention is diluted

Adams JL, Davis AC, Schneider EC, Hull MM, and McGlynn EA.

Article DOI: 10.1111/1475-6773.14014

APPENDIX A

A proof-of-concept simulation for the Distillation Method

The Distillation Method is an attempt to estimate a "post hoc" set of eligibility criteria that have a larger average treatment effect (ATE) than the original randomized trial population. Restricting the predictors used to pre-randomization covariates prevents selection on unobservable covariates from influencing who is included in the distilled analysis. Using treatment uptake, be it enrollment or an effective dose criterion, as a proxy for treatment effect in the uptake model avoids using outcomes. Much like propensity score methods, avoiding the use of outcomes prevents incorporating selection on unmeasured confounders into the revised eligibility criteria. This prevents the threat to validity inherent in as treated or per protocol analyses.

It is useful to distinguish here between an estimand, an unknown quantity of interest and an estimator, a data summary. In a typical evaluation of a proposed method, the estimand is a fixed unknown quantity, for example, the hypothetical mean treatment effect in the study population. The estimator would be the calculated value from the data (e.g., the average of the outcome in the treatment arm minus the average of the outcome in the control arm).

The Distillation Method is different from most estimation methods in that it does not fix the estimand as a fixed *a priori* quantity. Rather it jointly attempts to select an estimand and an

estimator that maximizes statistical power. The estimand is still an ATE but it is an ATE for a subset of the population in the original RCT. The estimator is still a typical treatment effect estimator but applied to a subset of the participants in the RCT. Conceptually the method is a very ordinary estimation approach applied to an empirically defined population that is a subset of the original RCT population.

The additional assumptions the Distillation Method requires are that there be a relationship between treatment uptake and the magnitude of the treatment effect and there is at least one pretreatment predictor of treatment uptake. Although we motivate the plausible existence of such cases in this manuscript by discussing motivations of subjects and recruiters the method only requires these properties. The method is not dependent on the relationships being generated by this posited mechanism.

As implemented here, failing to satisfy these assumptions has the harmless property of defaulting to the original ITT estimator. In the simulations presented below the estimator simply returns the original ITT results and the method may be abandoned. Note also that the method does not seem to increase the type I error rate in the cases where there is no treatment effect to be found. Under the assumptions used to generate the simulation data there does not seem to be any danger of using the method to capitalize on chance or engage in "p hacking." Note that if there is no correlation between the probability of uptake and treatment effect or there are no predictors of uptake the estimands of the distillation estimate and the original ITT estimate are the same. This is the situation where a Hausman specification test[1] is straightforward. Regardless of the treatment effect estimator employed the difference between the estimates for ITT and distillation is a test statistic. Because these estimates will in general be correlated a standard error estimate for the difference would generally require a bootstrap calculation. Bootstrap standard errors also

free the analyst to select the most appropriate statistical model for the outcomes. This approach can be applied if the outcome model is a cost model or a logistic regression or any other model of interest. In practice a practical difference between the estimates with a small enough standard error for the distillation estimate should suffice.

Simulation design

The above discussion suggests that the simulation must, at a minimum, have three underlying variables and a mechanism to correlate them. The three quantities required: 1) a treatment effect, 2) an uptake effect, and 3) a predictor of uptake. For simulation purposes the correlation between the variables will be induced by generating them from a trivariate normal distribution. The framework is similar in form to the probit style mechanisms used to study limited dependent variables. See, for example, Maddala[2]. The simulation study below is designed as a 6 by 4^3 by 11^2 factorial design with 6 levels of treatment effect, 4 levels of pairwise correlation between the variables in the generating trivariate normal, and 11 values of both the uptake rate and the refractory fraction. Several other potentially variable parameters (e.g. sample size) are set to single values that correspond to the features of the example. These values are discussed below. Researchers applying the Distillation Method may wish to run their own customized version of these simulations. This would be particularly useful for more complicated second stage models like two-part cost models. The simulation code is available from the authors.

Treatment effect:

A person level (heterogenous) treatment effect and outcome:

We start with a "per protocol" person level heterogeneous treatment effect. This is the benefit, in expectation, that would occur if the subject took the treatment.

τ_i= τ_{max} if X_{τ_i}>*refractory*, 0 else.

Where τ_{max} is set by us: (0,-0.1,-0.2,-0.3,-0.4,-0.5). Note zero is the traditional null hypothesis. The zero case allows us to check for any possible multiple testing or p-hacking effects. And *refractory* is the fraction of the population that gets no benefit from the treatment. The value of refractory is set to run from 0 to 1.0 by 0.1 in these simulations. The treatment effect heterogeneity in this model is a simple binary model where the refractory fraction of the population would receive no benefit of uptake and the nonrefractory fraction of the population receives τ_{max}. Normally distributed or other treatment effect distributions could be accommodated in a similar manner. The generation process for X_{τ_i} is defined below.

Uptake effect:

The subjects in the treatment group only receive the treatment effect if they take the treatment. Their actual treatment effect is:

$\tau actual_i$ = τ_i if Uptake=1, 0 else.

This is the hypothetical treatment effect. The observed outcome adds a normal error term:

Y_i= N($\tau actual_i$, σ^2)

Where σ^2 is 1.0 This is meant to mimic a log normal cost analysis. If the interest where to model a binary outcome a probit generating process could be substituted here. Even more elaborate generating processes are possible.

A person level uptake effect:

Uptake = 1 if $Puptake_i$ >(1 - UptakeRate), 0 else.

In these simulations we used values of UptakeRate from 0 to 1.0 by 0.1. The $Xuptake_i$ generation process is defined below.

A predictor of upt ake:$Xpred_i$ is a normal random variable. This represents the predictions of the first stage uptake model. Regardless of the functional form of the model it is the correlations between the predictions from the model and the uptake and treatment effects that produces the distillation effect.

The joint distribution generating mechanism:

The joint distribution of X_{τ_i}, $Xuptake_i$, and $Xpred_i$ is trivariate normal:

$$\begin{bmatrix} X_{\tau_i} \\ Xuptake_i \\ Xpred_i \end{bmatrix} \sim \mathrm{N}\left(\begin{bmatrix} 0 \\ 0 \\ 0 \end{bmatrix}, \Sigma \right)$$

Where:

$$\Sigma = \begin{bmatrix} 1 & \rho_{\tau u} & \rho_{\tau p} \\ \rho_{\tau u} & 1 & \rho_{up} \\ \rho_{\tau p} & \rho_{up} & 1 \end{bmatrix}$$

And the ρ are from $\{0,0.3,0.6,0.8\}$. Note that these are the generating correlations, not the observed correlations in the data. The dichotomization process will result in lower empirical correlations.

The data sets have sample sizes of 400 in both treatment and control. This value was selected to match the example.

Analysis of simulation results

The analysis of the simulations is challenging due to the complicated interactions between the simulation input values and their effects on power. We began with a logistic regression model with the largest power across all cut points, including the original full data set ITT analysis, as the dependent variable. We used an offset term in the model of the full data analysis power. This term is: ln(PowerITT/(1-PowerITT)). This results in a model for the increase in power from picking the most powerful cut point for the data set. As the independent variables we included all of the simulation design parameters and all of the interaction terms up to 6-way. We removed the 6-way interaction as non-significant but several 5-way interaction terms were significant. This precludes making simple statements about the effects of the various simulation input parameters one at a time. However the correlations between the input parameters and the power increase has the intuitive signs: abs(τ_{max}) (0.45), UptakeRate (0.16), Refractory (-0.16), $\rho_{\tau u}$ (0.06) $\rho_{\tau p}$ (0.49), ρ_{up} (0.49).

Figure A.1 helps visualize the relationships between full data set power and distilled power. The datapoints are a 10% sample of the predicted power from the 5-way interaction model. The curved line is a LOWESS curve using all of the data. By construction the maximum power is always greater than or equal to the full dataset power. For values of the full dataset power in the 0 to 20% range the Distillation Method can increase power but typically not enough to get to the traditional power thresholds of 80% or greater. So the power range from 20% to 80% are the most useful for distillation. Within this range more favorable relationships between the correlations and the refractory fraction correspond to greater power increases.

At first consideration it may seem that these results would suggest that the Distillation Method would only be useful in a narrow range of circumstances. If the ITT power is high enough the Distillation Method may not be needed. If the ITT power is low enough the Distillation Method

cannot help. But it is precisely the case where the power has slipped below anticipated *a priori* assumptions from the original power calculations that distillation offers the most potential improvement.

Figure A.1: Distillation Power vs. Original ITT Power

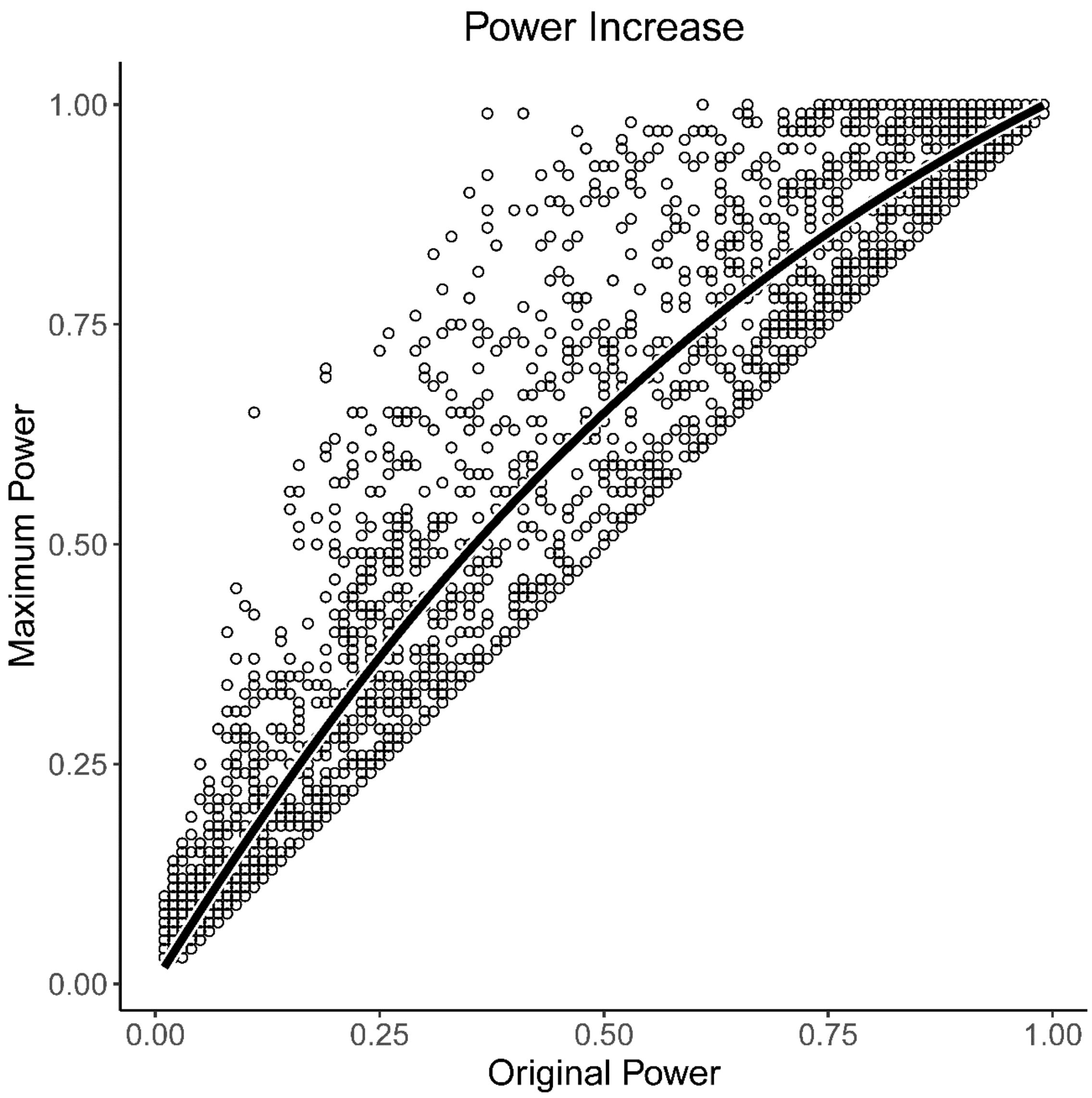

References

1. Hausman JA. Specification tests in econometrics. *Econometrica: Journal of the econometric society*. 1978:1251-1271.
2. Maddala GS. *Limited-Dependent and Qualitative Variables in Econometrics*. Econometric Society Monographs. Cambridge University Press; 1983.
