# Supplementary material for: The distillation method: A novel approach for analyzing randomized trials when exposure to the intervention is diluted
Source: Health Serv Res. 2022 Jul 19;57(6):1361–9. doi: 10.1111/1475-6773.14014 (PMC9643092; doi:10.1111/1475-6773.14014)
Supplement: Supplementary file 2 — Data S2. Supporting information. [file HESR-57-1361-s002.pdf]

The Distillation Method: A novel approach for analyzing randomized trials when exposure to the intervention is diluted

Adams JL, Davis AC, Schneider EC, Hull MM, and McGlynn EA.

Article DOI: 10.1111/1475-6773.14014

APPENDIX B

Additional Results of the Case Study

Figure B.1. Model performance for predicting intervention uptake in an RCT of a complex case management intervention.

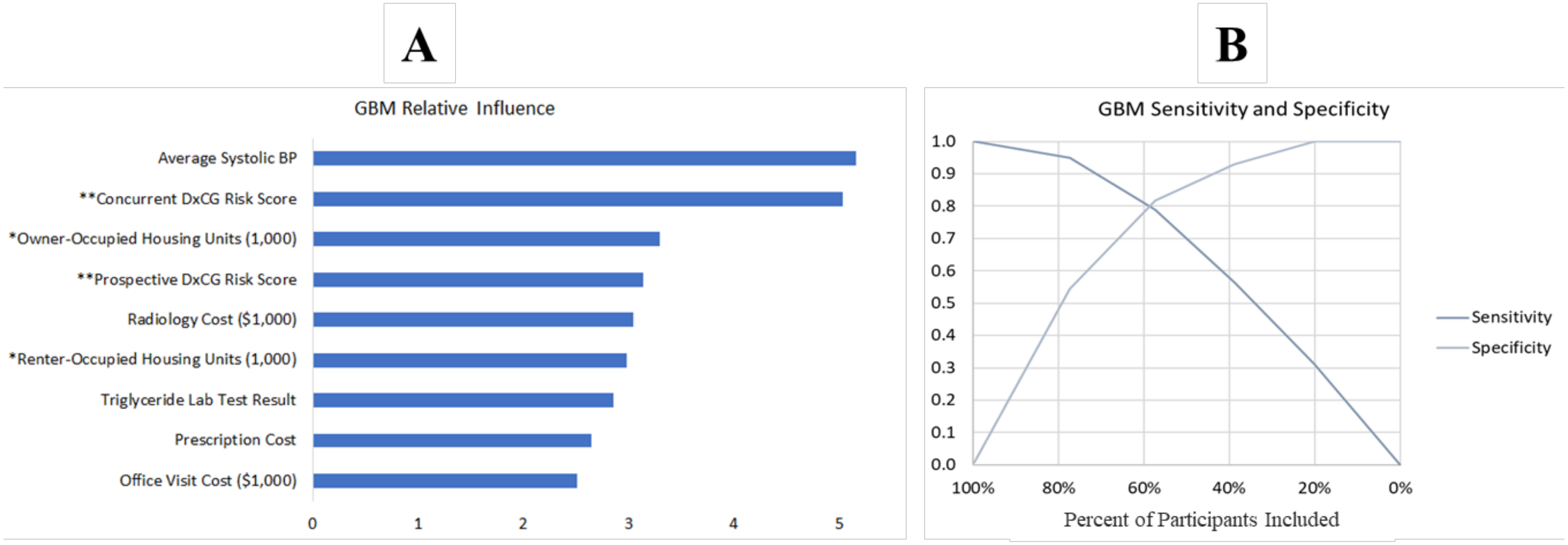

* Census-based variable

** Risk score

Figure presents the performance of the stage one predictive model for intervention participation in the pilot RCT examined as a case study for the Distillation Method. Panel A highlights the top 10 influential predictors showing relative influence in the Generalized Boosted Regression Models; because interpretation of this measure is relative, values of the influence measure are not shown. Panel B provides the sensitivity and specificity of the model at various levels of distillation, displayed as the percent of participants included in analysis.
